# Supplementary material for: Effects of Sulforaphane-Induced Cell Death upon Repeated Passage of Either P-Glycoprotein-Negative or P-Glycoprotein-Positive L1210 Cell Variants
Source: Int J Mol Sci. 2022 Sep 16;23(18):10818. doi: 10.3390/ijms231810818 (PMC9501161; doi:10.3390/ijms231810818)
Supplement: Supplementary file 1 [file ijms-23-10818-s001.zip › ijms-1908845-supplementary.pdf]

International Journal of
Molecular Sciences

MDPI

Supplementary files

Effects of sulforaphane-induced cell death upon repeated passage of either P-glycoprotein-negative or P-glycoprotein-positive L1210 cell variants

Anna Bertová et al.

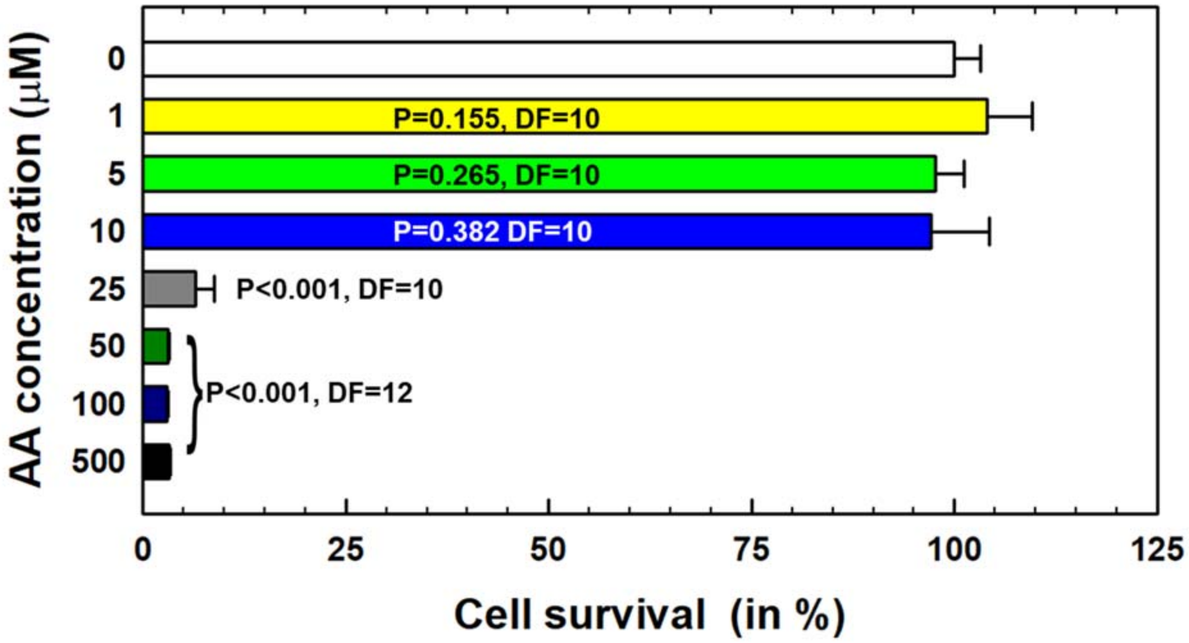

Figure S1. Effect of ascorbic acid (AA) on parental S cell viability. S cells were cultured under standard conditions in the presence or absence of AA. After cell culture, the MTT assay was used to estimate cell viability. The results represent the mean values ± SD. Changes were considered significant when P<0.05.

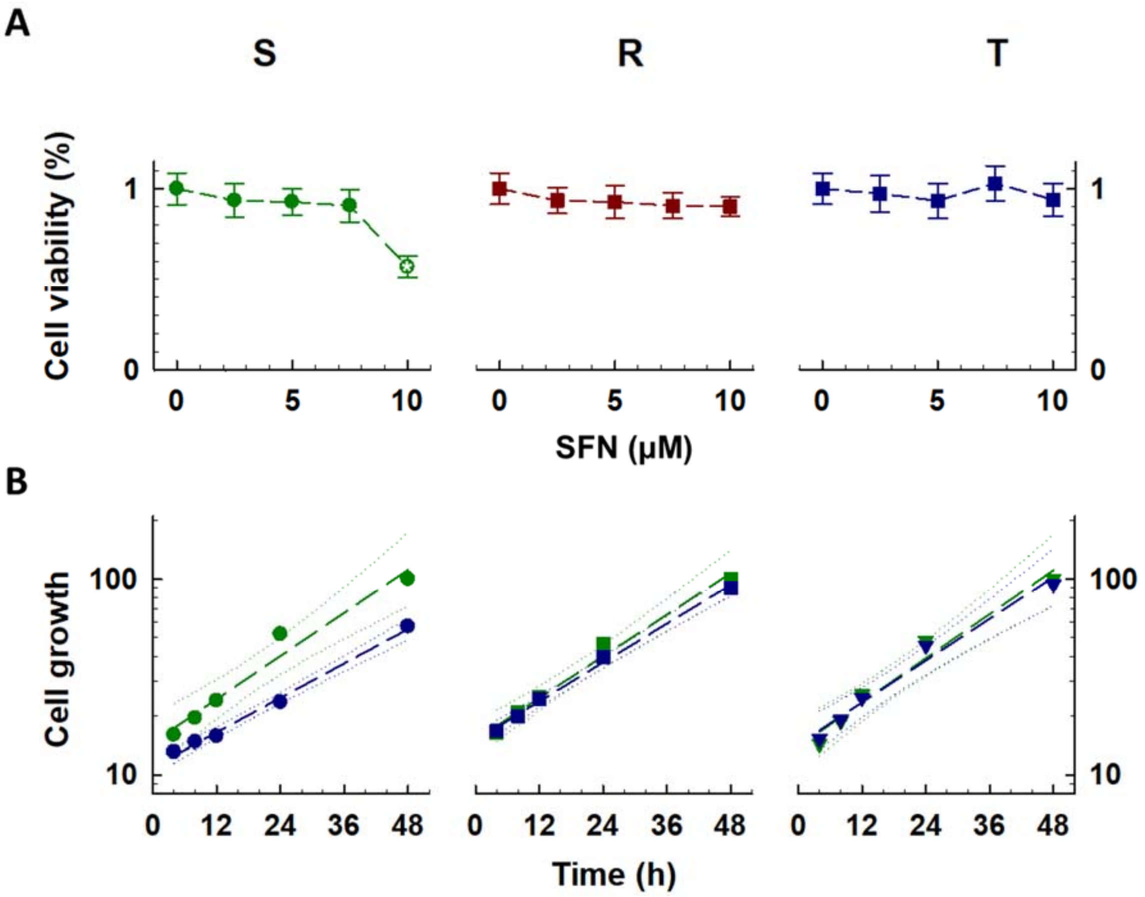

Figure S2. Effect of preincubation of S, R and T cells in the presence of SFN on viability and cell growth after subsequent culture in the absence of SFN. Panel A: Viability of S, R and T cells after the cultures described above. Data are expressed as % of control in the absence of SFN and represent the mean of six independent measurements. * - significantly different from the control in the absence of SFN at the level of P<0.02. Panel B: Kinetics of L1210 cell variant proliferation. The time courses of proliferation can be described by first-order kinetics for each cell variant (as we similarly documented elsewhere [69]) and therefore give a straight line in the semilogarithmic plot. Each point represents the average of six independent values, which were all used for correlation. Solid line: regression line; dashed line: 99% confidence interval valid for 28 degrees of freedom.Green - cells were precultured in the absence of SFN; blue - cells were precultured in the presence of 10 µM SFN.

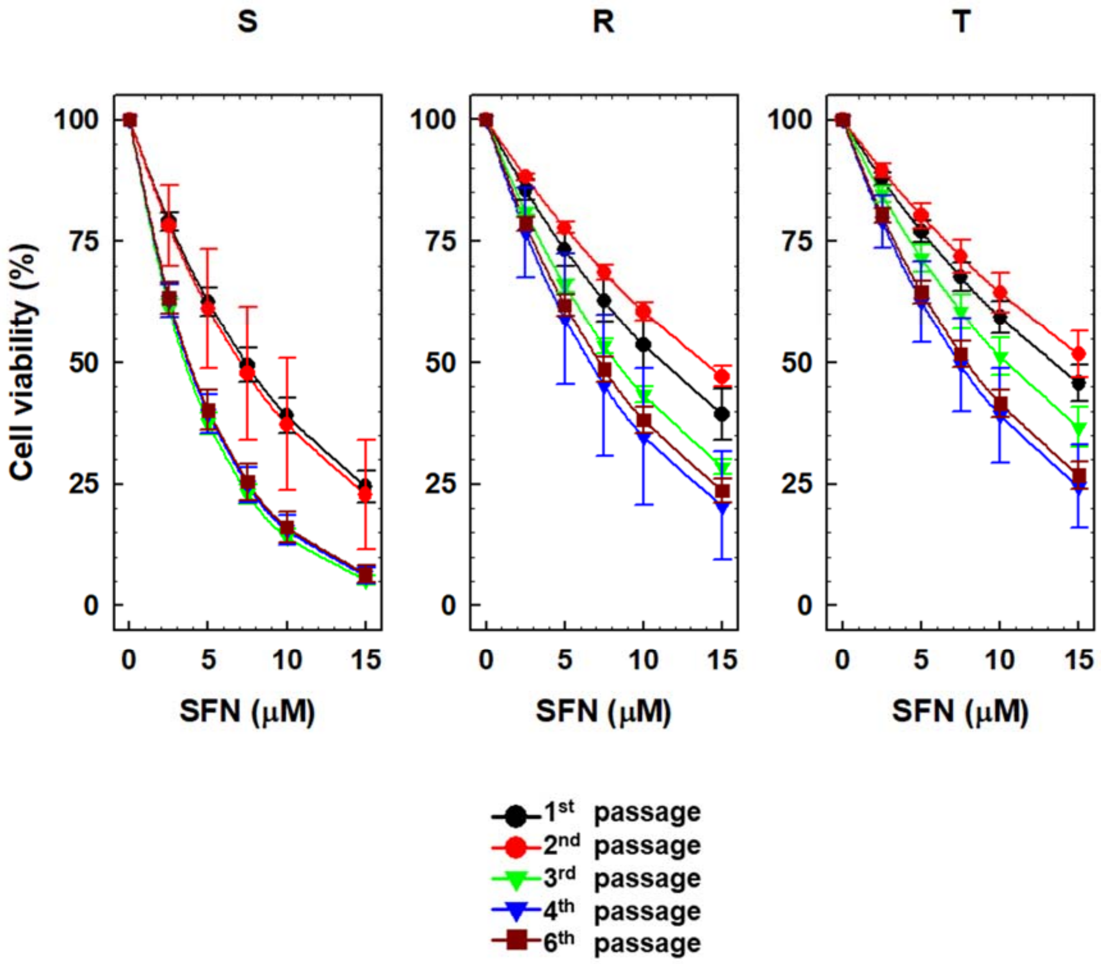

Figure S3. The number of S, R and T cells after individual passages in the presence of SFN at the indicated concentration determined by direct counting of viable cells in the CASY Model TT-Cell Counter. Data are expressed as % of control in the absence of SFN and represent the mean±SD of nine independent measurements. The experimental data were fitted according to Eq. 1 (Section 4.4), and the estimated IC_{50} values are summarized in Figure 4 (Section 2.3).

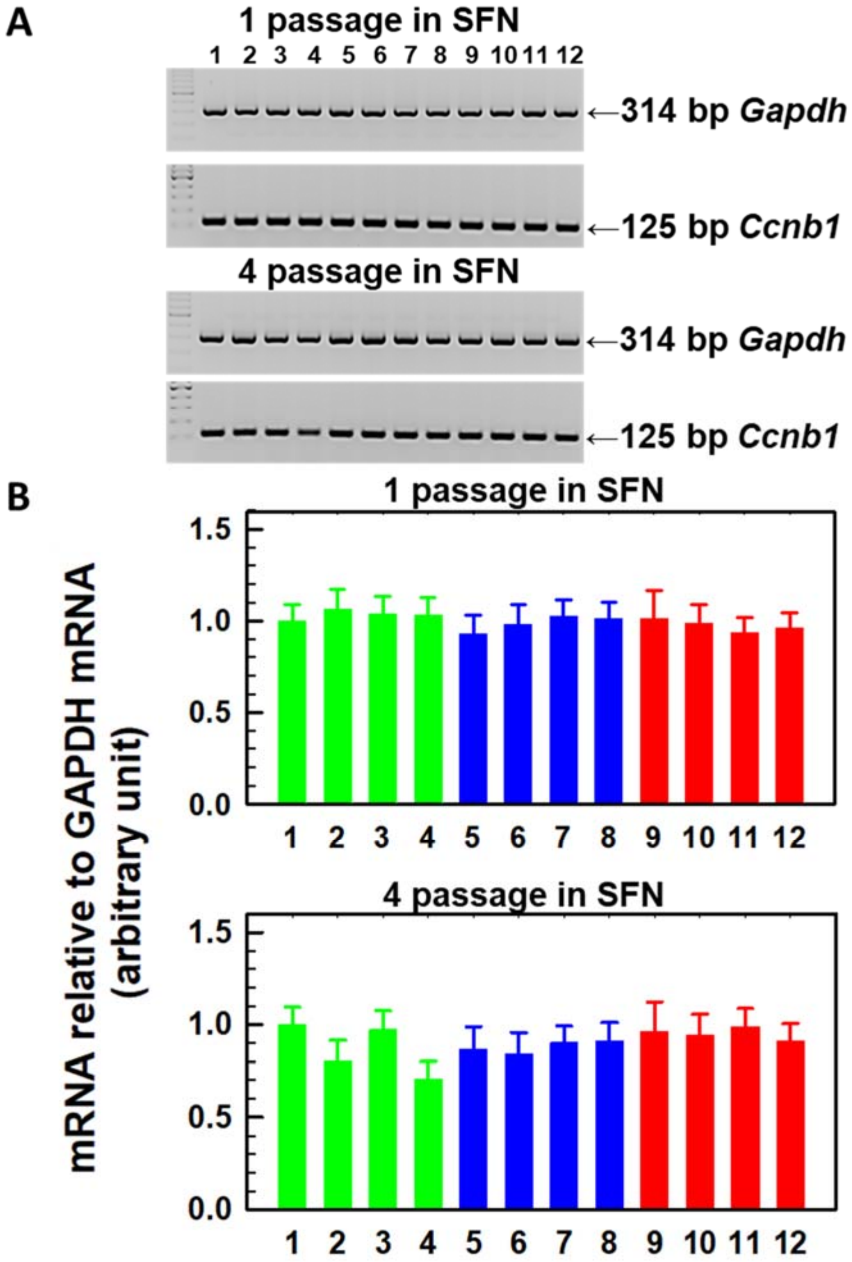

Figure S4. Changes in the relative gene expression of *Ccnb1* (for cyclin B1) in S, R, and T cells passaged either once or four times in medium containing SFN. At each passage, cells were cultured for 48 h in the presence of SFN at the following concentrations: 1 - 0.0 µM S cells; 2-5 µM S cells; 3-10.0 µM S cells; 4-15 µM S cells; 5 - 0.0 µM R cells; 6-5 µM R cells; 7-10.0 µM R cells; 8-15 µM R cells; 9 - 0.0 µM T cells; 10 - 5 µM T cells; 11 - 10.0 µM T cells; 12-15 µM T cells. Panel A: Electrophoretic analysis of the respective PCR products. Data represent three independent measurements. The *GAPDH* gene was used as an internal control. Panel B: The optical densities of the PCR product bands were quantified and are summarized in bar graphs. Data are expressed as the mean ± SD of three independent measurements. S cells (green), R cells (blue) and T cells (red). No significant changes in Ccnb1 expression were observed.

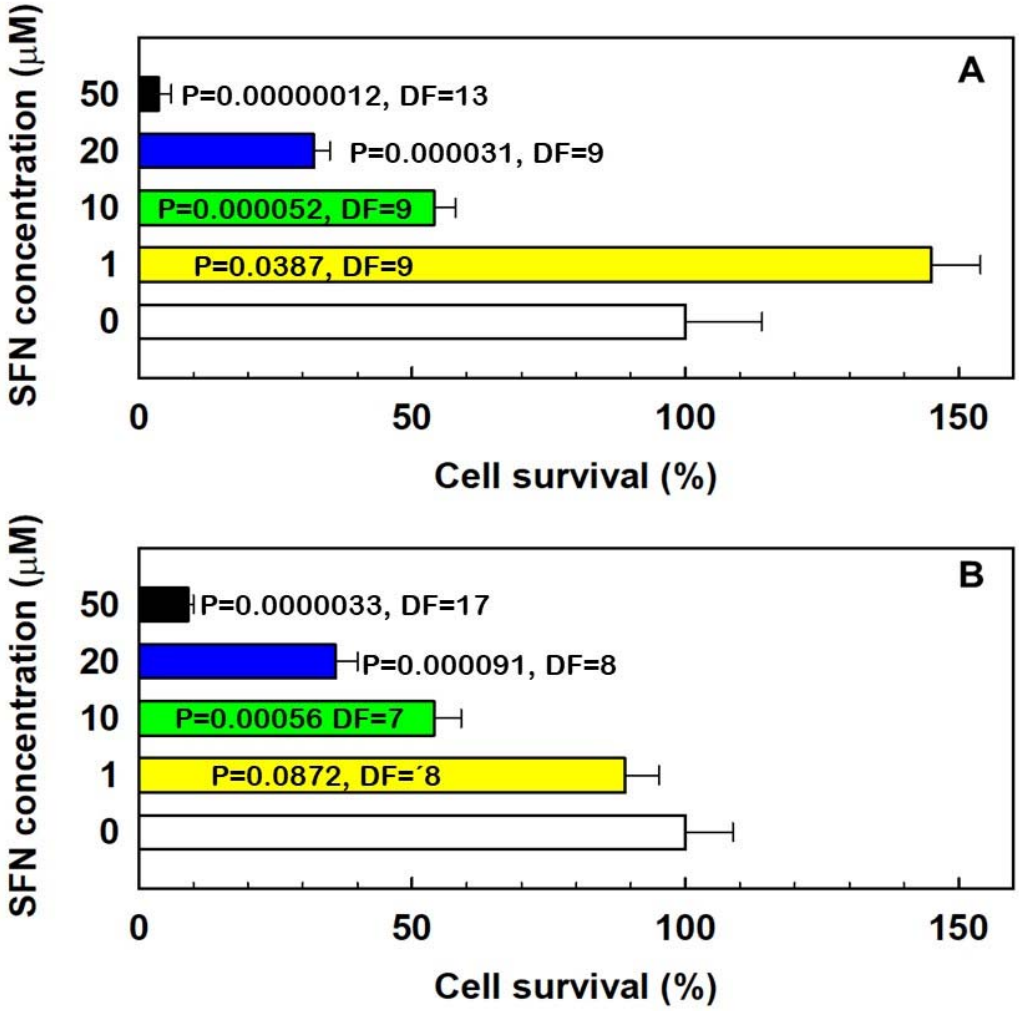

Figure S5. Concentration dependence of sulforaphane-induced cell death effects on S cells. S cells were cultured under standard conditions in the presence or absence of SFN at the indicated concentration. After culturing the cells were either subjected to in MTT assay (Panel A) or were directly counted in CASY Model TT-cell counter (Panel B). The results represent mean ± SD. Changes were considered significant when $P<0.05$.

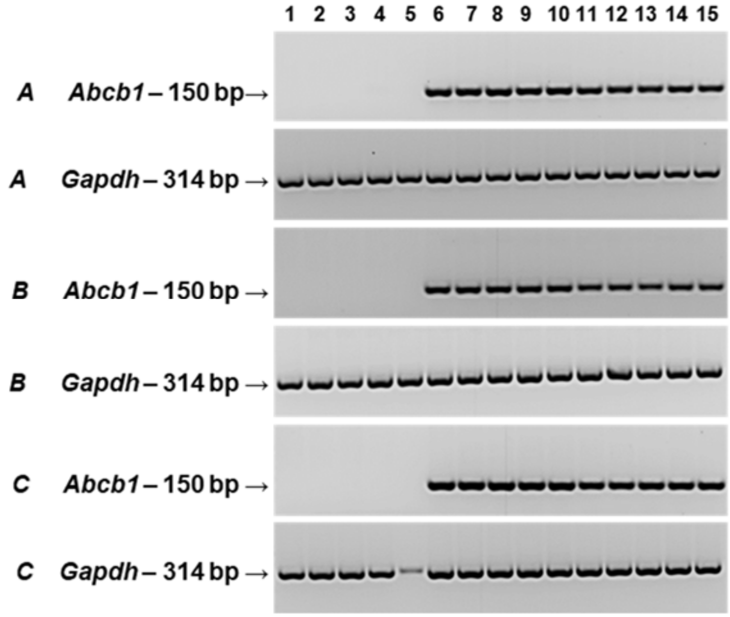

Figure S6. Changes in relative gene expression of Abcb1 (for ABCB1 transporter) in S, R and T cells passaged in the presence of SFN once (A); once and subsequently once in the absence of SFN (B); six times (C). At each passage, cells were cultured for 48 hours in the presence of SFN at the following concentrations: 1 - 0.0 μM S cells; 2 - 2.5 μM S cells; 3 - 5.0 μM S cells; 4 - 7.5 μM S cells; 5 - 10.0 μM S cells; 6 - 0.0 μM R cells; 7 - 2.5 μM R cells; 8 - 5.0 μM R cells; 9 - 7.5 μM R cells; 10 - 10.0 μM R cells; 11 - 0.0 μM T cells; 12 - 2.5 μM T cells; 13 - 5.0 μM T cells; 14 - 7.5 μM T cells; 15 - 10.0 μM T cells. PCR products were visualized electrophoretically. Data represent three independent measurements. The GAPDH gene was used as an internal control. The results show that there is no induction of the ABCB1 transporter in S cells or visible changes in its expression in R and T cells.
